# Plate waste and intake of school lunch based on the new Nordic diet and on packed lunches: a randomised controlled trial in 8- to 11-year-old Danish children

**DOI:** 10.1017/jns.2015.3

**Published:** 2015-05-06

**Authors:** Anne V. Thorsen, Anne D. Lassen, Elisabeth W. Andersen, Lene M. Christensen, Anja Biltoft-Jensen, Rikke Andersen, Camilla T. Damsgaard, Kim F. Michaelsen, Inge Tetens

**Affiliations:** 1 Division of Nutrition, National Food Institute, Technical University of Denmark, Søborg, Denmark; 2 Department of Applied Mathematics and Computer Science, Technical University of Denmark, Søborg, Denmark; 3 Department of Nutrition, Exercise and Sports, Faculty of Science, University of Copenhagen, Copenhagen, Denmark

**Keywords:** Edible plate waste, School meals, Packed lunches, Food liking, NND, new Nordic diet, OPUS, optimal well-being, development and health for Danish children through a healthy new Nordic Diet [English translation]

## Abstract

The aim of the present study was to compare total food intake, total and relative edible plate waste and self-reported food likings between school lunch based on the new Nordic diet (NND) and packed lunch from home. In two 3-month periods in a cluster-randomised controlled unblinded cross-over study 3rd- and 4th-grade children (*n* 187) from two municipal schools received lunch meals based on NND principles and their usual packed lunch (control). Food intake and plate waste (*n* 1558) were calculated after weighing lunch plates before and after the meal for five consecutive days and self-reported likings (*n* 905) assessed by a web-based questionnaire. Average food intake was 6 % higher for the NND period compared with the packed lunch period. The quantity of NND intake varied with the menu (*P* < 0·0001) and was positively associated with self-reported likings. The edible plate waste was 88 (sd 80) g for the NND period and 43 (sd 60) g for the packed lunch period whereas the relative edible plate waste was no different between periods for meals having waste (*n* 1050). Edible plate waste differed between menus (*P* < 0·0001), with more waste on soup days (36 %) and vegetarian days (23 %) compared with the packed lunch period. Self-reported likings were negatively associated with percentage plate waste (*P* < 0·0001). The study suggests that portion sizes need to be considered in new school meal programmes. New strategies with focus on reduction of plate waste, children's likings and nutritious school meals are crucial from both a nutritional, economic and environmental point of view.

Children spend more time in schools than in any other environment away from home and while education is the primary role of the schools, the schools cannot entirely achieve their mission if children are not alert and ready to learn which are related to a healthy diet and physical activity^(^^1^^)^. Additionally, schools are important settings to improve access to healthier foods for preventing overweight, obesity and chronic diseases in the long term and to reach children across all ethnic and socio-economic groups^(^^1^^–^^3^^)^. Several school-based interventions have documented that it is possible to influence school children's dietary habits positively^(^^2^^–^^4^^)^.

The majority of Danish children do not eat in accordance with the national dietary guidelines, but consume too many energy-dense and nutrient-poor foods, too much sugar, salt, saturated fat and fat, and too little wholegrain, fruits, vegetables and fish^(^^5^^–^^8^^)^. Furthermore, the social differences in overweight and obesity have widened from 2000–2002 to 2005–2008, especially for boys of parents with a short education^(^^9^^)^.

Denmark has no national school food programme and three out of four Danish children bring a packed lunch from home as only a few schools have canteens^(^^5^^)^. Typically, a packed lunch consists of open-faced sandwiches on Danish-style rye bread with liver paste or other types of meat products and sometimes vegetables and fruit^(^^5^^)^. The OPUS School Meal Intervention was conducted comparing new Nordic diet (NND) meals with packed lunches from home in order to investigate the health impact of serving school meals based on the NND^(^^10^^)^. Translated into English, OPUS is an abbreviation for ‘optimal well-being, development and health for Danish children through a healthy new Nordic Diet’.

Fundamental for NND dietary principles are that meals are palatable, environmentally friendly and largely based on food originating from the Nordic region^(^^11^^)^. Another key principle for the NND is to obtain less waste from the overall food production which includes an appropriate food intake as well as minimising food waste because food wasted affects not only the cost of the meal but also the climate footprint^(^^11^^–^^13^^)^.

Plate waste is generally defined as the quantity of edible food served that is uneaten^(^^14^^)^. Some plate waste is unavoidable, given the diversity of children and daily variation in appetite but excessive plate waste may indicate unnecessary costs of a meal programme and furthermore plate waste may have negative impact on the climate footprint^(^^12^^,^^14^^,^^15^^)^. Additionally, the potential health effects of the NND in a free-living population are dependent on the NND meals being actually eaten and not just served.

The measurement of food waste of different school lunch formats is new – and timely with the current awareness of food waste overall in a dietary sustainability context. Furthermore, to our knowledge, no randomised controlled trial has assessed the impact of introducing a whole new full meal concept covering the whole lunch, while at the same time performing careful measurements of children's plate waste.

The aim of the present study was to investigate whether the amount of food intake and total and relative edible plate waste differed between packed lunches from home and school meals served based on NND principles. A further objective was to examine how food intake and food waste at the two types of meals are associated with the children's likings of the meals.

## Methods

### Study design and recruitment

The OPUS School Meal Study is a cluster-randomised controlled unblinded cross-over study. In two 3-month periods 3rd- and 4th-grade children (aged 8–11 years) from nine selected municipal schools received school meals based on the NND and their usual packed lunch (control) in random order during the school year 2011–2012. A number of measurements were performed before the start of the first dietary period (baseline), at the end of the first dietary period (month 3) and at the end of the second dietary period (month 6). The study design and recruitment to the OPUS School Meal Study including this plate waste study have previously been described in detail^(^^10^^)^.

Two of the nine selected schools were randomly assigned to the present study of food intake and plate waste. The NND meals were produced locally at each school by trained chefs and kitchen personnel hired for the study and small groups of children participated in the cooking every day.

The packed lunches were eaten in the classrooms whereas the NND meals were prepared and served in a cafeteria set up for the purpose of the study. Supervised by the OPUS kitchen personnel, four to six alternating pupils served the *ad libitum* hot NND meals at the buffet. The children were encouraged to taste all food served and keep a reasonable plate distribution with vegetable and starchy food filling the majority of the plate^(^^16^^)^. The adult kitchen personnel supervised four to six alternating children who helped to serve the NND meals.

The guidelines and dietary composition and nutrient content of NND have been described in detail by Mitril *et al.*^(^^11^^,^^17^^)^. The NND menus contained more berries, cabbage, root vegetables, legumes, fresh herbs, potatoes, wild plants and mushrooms, whole grains, nuts, fish, shellfish and less meat than the average Danish diet^(^^10^^,^^11^^,^^17^^)^. The energy density of the NND lunch was on average 560 kJ/100 g whereas the energy density of the packed lunch on average was 740 kJ/100 g.

A 3-week NND menu was developed and served for each of the three seasons (autumn, winter and spring). The menu plan for the NND meals were the following: Monday: soup and some fruit for dessert; Tuesday: meat; Wednesday: vegetarian dish and a dessert; Thursday: fish; Friday: buffet-style with calculated leftovers from the menu every day, Monday to Thursday. Each weekly schedule had a balance between different categories of ingredients consistent with the nutritional recommendations, and took into account the feasibility in preparing the meals and incorporating calculated leftovers from the menu every day, Monday to Thursday, to be utilised on Fridays. Tap water was served and available at the tables with the NND lunch meals.

The NND meals were free of charge for all 3rd- and 4th-grade children at the participating schools. The lunch break was 20 min when having a packed lunch but increased to 25 min at school A and from 25 min up to 40 min at school B when getting the NND.

### Food intake and edible plate waste

The amount of food intake and edible plate waste at lunch were measured for five consecutive days by weighing each child's lunch before and after eating. To ensure that the children had time to get used to the new NND diet the measurements were taken at the end of the intervention period. The method used was developed by Sabinsky *et al.*^(^^18^^)^ and adapted by Thorsen & Biltoft-Jensen^(^^19^^)^. Beverages were not included in this study. Food intake and edible plate waste = 5 g were regarded as no intake or no waste.

### Recording food intake and edible plate waste for packed lunch from home

Before lunch break solid polystyrene plates and trays tagged with class, date and identity numbers were distributed to all children in the classrooms. The children were asked to unpack their packed lunches from home, place their food on the plate and to separate items and open up the sandwiches, so all food items would be observable. The children brought their food to the weighing station outside the classroom where a trained assistant weighed the plate (Vera 67002, with a precision of ± 1 g; Soehnle) and then to the photograph station where another trained assistant took a photograph of the plate (Nikon COOLPIX S 210 digital camera). When the children had finished eating the procedure was repeated. Any package or wrapping that was weighed the first time (yoghurt and noodle cups, muesli bar wrappings, etc.) was left on the plate and also weighed the second time. If the plate waste also included non-edible food items (fruit peel, wrapping, etc.) these were removed and the plate and edible waste were weighed again. The photographs were used for confirming the weight data. The weights of the plates were subtracted from the pre- and post-measures and also the served weight was adjusted for non-edible items. The relative edible plate waste was also calculated by comparing the edible plate waste with the total amount of edible food served.

### Recording food intake and edible plate waste for the new Nordic diet lunch

The same procedure as described above for packed lunches brought from home was used except that the measurements were taken near the buffet. If the children chose to have a second serving the procedure was repeated and the NND food was weighed and photographed before and after eating.

### Self-reported smiley rating from the Web-based Dietary Assessment Software for Children

During the OPUS School Meal Study, dietary assessment at baseline, and at dietary periods 1 and 2 were obtained from each child using the Web-based Dietary Assessment Software for Children (WebDASC)^(^^20^^)^. A self-reported smiley rating was included in the WebDASC to measure the children's liking of the lunch on a scale from 1 to 5 (1 = really bad, 2 = bad, 3 = okay, 4 = good and 5 = really good). Since only two packed lunches were rated really bad and two packed lunches were rated bad these two groups were combined.

### Background information

At baseline each child together with at least one parent or custody holder underwent a 2-h in-depth interview by a trained interviewer including instructions in using the dietary assessment tool, either at the school or at home^(^^10^^)^. The interview included background information like sex, age group and socio-economic status of the household. The educational level of the household was categorised according to the standard classifications of Statistics Denmark, i.e. as the highest level of education achieved by a parent in the household^(^^21^^)^. The variable was divided into six different groups depending on the educational level (lower secondary education; upper secondary education or equivalent; vocational education; short higher education; bachelor's degree or equivalent; and master's degree).

### Data analyses and statistics

Analyses included standard descriptive statistics. Two types of outcomes were analysed: binary (i.e. waste/no waste); and continuous (i.e. weight of food intake and edible plate waste). Data were therefore modelled in two steps with regard to edible waste: first, a logistic regression model with random effects fitted for the probability of waste/no waste; and second, a model fitted for either total food intake or total or relative edible plate waste data^(^^22^^)^.

To analyse the percentage edible plate waste (in lunches having edible plate waste) random-effects models were used for intake and waste (g). First a model was fitted with two random effects (child and class) to take the design into account. The model was also adjusted for school, sex, grade, dietary period, household education level and intervention.

Since the study had a cluster-randomised cross-over design, classes were randomised to receive the NND either in the first or in the second dietary period with five measurements on each child in each period. This data structure resulted in two random effects: a child effect and a class effect (the whole class is randomised together). Two schools were analysed in the present study and treated as a fixed effect for both classes and schools. All models included school, sex, year group and household education level as fixed effects in the analysis. The assumptions underlying the models were tested using residual plots and QQ plots. It was necessary to transform the continuous outcome of interest using the logarithm but the results are presented on the original scale. The assumptions behind the model to analyse percentage edible plate waste were tested using residual plots and it turned out that the square root transformation gave the best results. The results are, however, expressed on the original scale as differences in percentage waste^(^^23^^)^.

SAS version 9.3 (SAS Institute, Inc.) was used for all statistical analyses. The significance level was chosen as 0·05.

## Results

Table 1 shows the descriptive statistics of the 187 children from the two municipal schools participating in the present study, 48 % from the 3rd and 52 % from the 4th grade. Of the children, 45 % were boys and 55 % were girls. All in all, 1558 lunches were analysed.

**Table 1.** Descriptive statistics for the 187 children in the study consuming 1558 meals over the total time period

	Children		Meals		Food intake (g)				Edible plate waste (g)				Waste (%)			
	*n*	%	*n*	%	Median	IQR	Mean	sd	Median	IQR	Mean	sd	Median	IQR	Mean	sd
In total	187		1558		196	126–278	214	118	40	1–103	66	77	15	0·2–39	23	24
Sex																
Boys	84	45	691	44	200	125–282	222	131	40	1–99	65	76	15	0·2–38	23	24
Girls	103	55	867	56	194	126–273	208	107	39	1–106	66	78	14	0·2–39	23	24
Intervention																
NND			793	51	209	128–311	230	134	68	20–133	88	80	25	6·3–46	29	24
Packed lunch			765	49	188	125–253	198	97	2	0–69	43	67	0·9	0·0–29	16	22
School																
School A	80	43	617	40	168	103–238	179	100	62	0–138	84	87	28	0·0–48	29	26
School B	107	57	941	61	219	148–304	237	123	28	1–87	54	68	9	0·3–32	18	22
Year group																
3rd grade	90	48	746	48	213	142–304	234	126	35	1–99	63	76	13	0·3–36	21	23
4th grade	97	52	812	52	182	113–258	196	107	46	0–108	69	78	18	0·0–41	25	25

IQR, interquartile range; NND, new Nordic diet.

One child had no information about household education level and was excluded from the analyses.

### Food intake

The median and mean food intake, edible plate waste and percentage edible plate waste are shown in Table 1. The lunch intake for the children was 230 (sd 134) g during the NND period and 198 (sd 97) g during the control period (packed lunch) (Table 1). The mean portion size (the amount of food served) was 318 g for the NND compared with packed lunches (241 g). The food intake at school A was 179 (sd 100) g compared with school B (237 (sd 123) g) (Table 1).

Table 2 lists the descriptive statistics for liking of the different menus (*n* 905). Of the NND meals the leftovers, vegetarian and cake and meat all had high likings with more than 39 % of the children rating the meal as really good. Soup was liked the least among the NND menus being rated as really bad/bad (18 % meals). Regarding the packed lunch, 46 % of meals were rated as really good and only 1 % of meals were rated as really bad or bad.

**Table 2.** Descriptive statistics for likings of the new Nordic diet meals and packed lunch for 905 meals (Numbers and percentages)

	Really bad/bad		Okay		Good		Really good		
	*n*	%	*n*	%	*n*	%	*n*	%	In total
Soup and fruit slices	24	18·0	37	27·8	45	33·8	27	20·3	133
Meat	8	7·1	21	18·6	40	35·4	44	38·9	113
Vegetarian and cake	5	4·0	21	17·0	47	37·9	51	41·1	124
Fish	8	7·3	23	18·5	42	38·2	37	33·6	110
Leftovers	1	1·0	24	23·3	35	36·1	43	41·7	103
Packed lunch	4	1·2	38	11·8	133	41·3	147	45·7	322
Total	50	5·5	164	18·1	342	37·8	349	38·6	905

Table 3 (model 1) shows that lunch intake was on average 6 % larger (g/lunch) (95 % CI 1·01, 1·12) for children having the NND compared with the period when they had packed lunch (*P* = 0·02) (*n* 1543). The NND lunch intake varied with the menu (*P* < 0·0001) (Table 3, model 2). When having soup the children had on average a 22 % higher lunch intake (95 % CI 1·12, 1·32) compared with packed lunches. Also on the vegetarian day the food intake was 38 % higher (95 % CI 1·26, 1·51) whereas the lunch intake was 14 % lower (95 % CI 0·79, 0·94) on the fish day compared with packed lunches.

**Table 3.** Results from three linear mixed models for the effect of the new Nordic diet (NND) on lunch intake* (Estimates and 95 % confidence intervals)

	Estimate	95 % CI	Test for no effect: *P*
Model 1 (*n* 1543)			
NND	1·06	1·01, 1·12	0·0229
Packed lunch	1		
Model 2 (*n* 1543)			
Soup	1·22	1·12, 1·32	<0·0001
Meat	1·00	0·91, 1·10	
Vegetarian and cake	1·38	1·26, 1·51	
Fish	0·86	0·79, 0·94	
Leftovers	0·93	0·85, 1·01	
Packed lunch	1		
Model 3 (*n* 1329)			
NND	1·13	1·07, 1·20	<0·0001
Packed lunch	1		
Lunch rating: really bad/bad	1		<0·0001
Lunch rating: okay	1·07	0·92, 1·26	
Lunch rating: good	1·40	1·21, 1·63	
Lunch rating: really good	1·59	1·36, 1·85	

Model 1, initial model for the effect of the NND; model 2, taking the menu into account; model 3, adjusting for liking.

* School, sex, year group, household education and dietary period were included as fixed effects in the analysis.

In Table 3 (model 3) the children's self-reported ratings of the lunch were compared with the lunch type (*n* 1329). The effect of the NND was increased when ratings were taking into account, so when sex, school, year group, household education, period and rating were included as fixed effects in the analysis the intake increased 13 % during the NND compared with the period with a packed lunch (95 % CI 1·07, 1·20) (Table 3, model 3). The amount of food intake increased with the rating of the lunch (*P* < 0·0001). If a lunch was rated really good the food intake was 59 % larger than when a lunch was rated as really bad or bad (95 % CI 1·36, 1·85).

### Edible plate waste

Looking at all lunch meals (*n* 1558) in Table 1, the edible plate waste was 88 (sd 80) g for the NND, resulting in a 29 % edible plate waste; for packed lunches the edible plate waste was 43 (sd 60) g, resulting in a 16 % edible plate waste.

A total of 1558 lunches were analysed and, of these, 498 lunches (26 %) had no edible plate waste. Of the 498 meals without edible plate waste, 102 (20·5 %) were NND meals and 396 (79·5 %) were packed lunches (results not shown). Also, children from the 3rd grade wasted 9 % less than children in the 4th grade after adjusting for school, sex, education, period and intervention (results not shown). The two schools performed differently; school A had 14 % more edible plate waste compared with school B (results not shown).

Table 4 (model 1) shows no significant difference between the NND and packed lunches considering only the plates having edible plate waste (*n* 1055) (*P* < 0·3506). However, Table 4 (model 2) shows that edible plate waste differed according to the menu; on the soup day the children had 36 % (95 % CI 1·15, 1·60) more edible plate waste than during the period with packed lunches. The vegetarian and cake menu day had significantly more waste (23 %) (95 % CI 1·04, 1·45) whereas there was less waste on the fish day (18 % decrease) (95 % CI 0·69, 0·97) compared with the period where the children consumed packed lunches. Plate waste on the leftover day and on the meat day was comparable with plate waste on packed lunch days.

**Table 4.** Results from three linear mixed models for the effect of the new Nordic diet (NND) on edible plate waste* (Estimates and 95 % confidence intervals)

	Estimate	95 % CI	Test for no effect: *P*
Model 1 (*n* 1055)			
NND	1·06	0·94, 1·19	0·3506
Packed lunch	1		
Model 2 (*n* 1055)			
Soup	1·36	1·15, 1·60	<0·0001
Meat	1·01	0·85, 1·20	
Vegetarian and cake	1·23	1·04, 1·45	
Fish	0·82	0·69, 0·97	
Leftovers	0·91	0·77, 1·08	
Packed lunch	1		
Model 3 (*n* 902)			
NND	1·01	0·89, 1·15	0·8586
Packed lunch			
Lunch rating: really bad/bad	1·69	1·29, 2·21	<0·0001
Lunch rating: okay	1·33	1·12, 1·59	
Lunch rating: good	1·05	0·92, 1·21	
Lunch rating: really good	1		

Model 1, initial model for the effect of the NND; model 2, taking the menu into account; model 3, adjusting for liking.

* School, sex, year group, household education and dietary period were included as fixed effects in the analysis for meals having edible plate waste.

Also, the amount of edible plate waste was negatively associated with the rating of the lunch as shown in Table 4 (model 3) (*P* < 0·0001). If a lunch was rated as really bad or bad then the plate waste on average was 69 % larger (95 % CI 1·29, 2·21) compared with a lunch rated as really good (Table 4, model 3).

Table 5 shows the relative edible plate waste (compared with the total amount of food served) for the NND and packed lunches. Table 5 (model 1) shows no significant differences between the percentage edible plate waste of the NND compared with packed lunches when looking at the plates having edible waste (*n* 1055) (*P* = 0·9280). No significant effect of sex was seen on percentage edible plate waste, but age group was highly significant on percentage plate waste (*P* < 0·0001) (results not shown).

**Table 5.** Results from three linear mixed models for the effect of the new Nordic diet (NND) on percentage edible plate waste* (Estimates and 95 % confidence intervals)

	Estimate	95 % CI	Test for no effect: *P*
Model 1 (*n* 1055)			
NND	–0·12	–2·64, 2·41	0·9280
Packed lunch	0		
Model 2 (*n* 1055)			
Soup	–0·12	–2·64, 2·41	0·1339
Meat	3·22	–0·38, 6·81	
Vegetarian and cake	–0·47	–4·36, 3·42	
Fish	–2·09	–5·76, 1·57	
Leftovers	–2·14	–5·98, 1·70	
Packed lunch	0		
Model 3 (*n* 902)			
NND	–1·78	–4·49, 0·93	0·1970
Packed lunch	0		
Lunch rating: really bad/bad	17·21	11·49, 22·92	<0·0001
Lunch rating: okay	11·83	8·09, 15·57	
Lunch rating: good	4·03	1·07, 6·99	
Lunch rating: really good	0		

Model 1, initial model for the effect of the NND; model 2, taking the menu into account; model 3, adjusting for liking.

* School, sex, year group, household education and dietary period were included as fixed effects in the analysis for meals having edible plate waste.

Looking at the different menus, the percentage waste (the relative plate waste) was 3 % larger on soup days (95 % CI –0·38, 6·81) than with packed lunches. The percentage edible plate waste did not differ significantly in the intervention groups (*P* = 0·1339).

Rating of lunch is highly significant for the percentage plate waste (*P* < 0·0001). If a lunch was rated as really bad or bad then the percentage plate waste was 17 % higher compared with a lunch rated as really good (95 % CI 11·49, 22·92) when including school, sex, year group, household education, dietary period and intervention as fixed effects (*n* 902) (Table 5, model 3).

As shown in Table 1 the average food intake for the NND was in fact higher (230 (sd 134) g/d) compared with packed lunches (198 (sd 97) g/d). Together with the higher edible plate waste in the NND period of 88 (sd 80) g compared with the packed lunch (43 (sd 67) g), this clearly shows an expected discrepancy. However, when the edible plate waste compared with the packed lunch was adjusted for the confounders the results showed clearly that the edible plate waste was significantly different for the meals where the meals with soup, vegetarian/cake, and fish were served (Table 4). These differences disappeared when the edible plate waste was expressed as a percentage of the served meal weight (Table 5). So the larger meals in weight and waste of the NND meals are taken into account.

## Discussion

In the present study the children receiving the NND had on average a 6 % larger lunch intake compared with the period when they had packed lunches. At the same time the average edible food waste was higher for the NND (mean 88 g) than for packed lunches (mean 43 g). Looking at the relative edible plate waste (comparing the edible plate waste with the total amount of food served) the children on average had 29 % edible plate waste when eating the NND compared with 16 % when eating packed lunches. This difference is mainly due to the fact that more children had edible plate waste in the NND group than in the packed lunch group. Among those having plate waste no significant difference was seen between having the NND and having packed lunches.

The soup day and the vegetarian and cake day were the days with the highest food intake and having the highest plate waste, indicating that the portion size needs to be adjusted for these meals.

When adjusting the portion sizes also the energy density of the food needs to be considered. In order to have an appropriate energy intake children need to eat bigger portion sizes of the NND having a lower energy density (in average 560 kJ/100 g) compared with packed lunches (740 kJ/100 g) which might be difficult for a child in a short lunch break and therefore resulting in a large plate waste for the NND compared with packed lunches^(^^16^^)^.

In a report on plate waste for Swedish school canteens the plate waste varied from 5 to 80 g per portion, depending on how much the children liked the food, measuring methods, conditions at the school and attitudes among the children and the staff^(^^24^^)^. Compared with the Swedish data the percentage edible plate waste for the NND (29 %; equivalent to 88 g) meal was high. However, the percentage edible plate waste varied according to the NND menu, with most waste on a soup day and the smallest amount of waste on fish day.

Also, Buzby & Guthrie^(^^14^^)^ found that plate waste varied with meal type, with vegetables and salad tending to be the most wasted items in the National School Lunch Program in the USA. The data were collected from 1991 to 1992 by 3350 students using a 24 h recall (no lunches from home). In that study, plate waste varied by age and sex; girls wasted more than boys. Children under 11 years of age wasted 15 % of their food while older children wasted less (11- to 14-year-olds wasted 12 %). In the present study we found no significant effect of sex on percentage edible plate waste, but age group was highly significant on percentage plate waste (*P* < 0·0001).

Baik & Lee^(^^25^^)^ found that school children (aged 6–9 years old) had plate waste from 15 to 19 % of the school lunch being served. In the present study the edible plate waste was 29 % for the NND, which is almost double the percentage compared with Baik & Lee's finding (15–19 %)^(^^25^^)^ and the finding by Buzby & Guthrie (12–15 %)^(^^14^^)^.

Bergman *et al.*^(^^26^^)^ found that meal scheduling could influence plate waste; students ate more and wasted less when eating lunch later during the school day and preferably after recess. In the present study the lunch break was longer when having the NND compared with packed lunches; up to 40 min lunch break at school B and 25 min at school A. This might explain part of the 6 % higher intake for the NND compared with packed lunches and also the higher food intake at school B compared with school A.

Liking school meals seems to be essential to reduce edible plate waste. In the present study the percentage edible plate waste was 17 % larger when the children rated the meal as really bad or bad compared with really good (*P* < 0·001). Maybe not surprisingly, packed lunches were rated higher than the different NND menus since parents normally would prepare a packed lunch that their children like (Table 2).

Nevertheless, an average percentage plate waste at 29 % for the NND is not sustainable compared with other school meal systems having 12–20 % plate waste^(^^14^^,^^24^^,^^25^^)^. On the other hand, NND meals are very different from packed lunch so it might take more than 3 months for the children to familiarise to the NND and to adjust portion sizes. The latter is supported by the amount of served food being almost 80 g larger for the NND (mean = 318 g) compared with packed lunches (mean = 241 g), which might be a result of the not adjusted portion sizes and not simply because the children did not like the NND meals. In order to make the NND meals sustainable it is crucial to ensure that the children eat in accordance with the dietary recommendations and guidelines including appropriate portion size.

Plate waste studies are expensive and time consuming in particular if the foods are weighed at the beginning and the end of a meal. Other methods such as visual estimates by trained observers or 24 h recalls by children do not take into account the actual plate waste^(^^14^^,^^15^^)^. Cohen *et al.*^(^^15^^)^ found the weighing method to be disruptive to normal lunch procedure and observed and estimated the waste instead. In the present study the setting of the NND meals was very different from the normal lunch procedure so it was decided to use the weighing method as this was considered the most accurate method.

One of the principles of the NND is to be environmentally friendly, and minimising edible plate waste is important because food wasted affects not only the cost of the meal but also the climate footprint^(^^12^^,^^15^^)^. When analysing the effect of climate optimising for the NND meals and the recommended Nordic diet these diets were found to be 20 % more climate friendly than the average Danish diet but these calculations did not include the 29 % edible plate waste for NND meals found in the present study^(^^12^^,^^13^^)^.

Interventions in real-life settings are complex systems that interact dynamically with the key stakeholders and therefore the intervention has to be tailored to the needs of the particular school environment in which it is implemented^(^^27^^–^^29^^)^. In the present study large differences were seen between the two schools and between the two year groups. One strategy to lower plate waste would be to tailor the intervention at the specific school taking into account the specific challenges at the school. Another strategy could be to network between the schools about the successes and challenges. Children from the 3rd grade wasted 9 % less than children in the 4th grade after adjusting for school, sex, education, period and intervention which is in contrast to the findings of Buzby & Guthrie^(^^14^^)^. The 4th graders seemed less guided by the teachers at lunch and often in a hurry to play, whereas the 3rd graders followed more willingly the guidelines of the teachers and the encouragements by chefs at the cafeterias. The two schools performed differently; school A had 14 % more edible plate waste compared with school B. One explanation for more plate waste could be that school A had a shorter lunch break than school B or maybe because the key stakeholders at school B took more ownership of the OPUS School Meal Study than at school A.

Serving smaller portion sizes would be another obvious strategy to reduce plate waste since food intake and plate waste were higher for the NND compared with packed lunch, however, this could result in insufficient energy intake for some children. In the present study it was seen that the 3rd graders were served larger portions than the 4th graders which might explain the 3rd graders higher food intake but not the lower 9 % lower plate waste (Table 1). Also Andersen *et al.*^(^^30^^)^ find that 3rd graders have a higher food intake than 4th graders in a study of all nine schools even though data were collected using a food record.

Strategies to reduce plate waste and getting children to like and eat a nutritious school meal would be interesting from not only a nutritional and economic point of view but also an environmental point of view. An earlier study of a Danish school-based meal programme showed that the nutritional quality of lunch was improved when the children had lunch provided by the school instead of packed lunch from home^(^^31^^)^. Also an American school cafeteria study showed an impact on the nutritional quality of the school meals when hiring a chef at the school cafeteria to make the meals not only nutritious but also palatable^(^^32^^)^. Another Danish school meal programme in Copenhagen (providing an average of 3700 meals daily) served healthy and tasty meals at a price that all children could afford in order to make equality in health by differentiating the price of the meal depending on the parents’ income level^(^^33^^)^.

A tailored long-term school lunch programme might be a way to get children in school to eat in accordance with the dietary guidelines including proper portion sizes and thereby diminishing food waste and maybe some of the socio-economics differences in food intake that are found among school-aged children.

The present study was designed in such a way that the 4th grade at school A received the NND in period 1 and the 3rd grade in period 2, while at school B it was the other way round. Therefore it was not possible to distinguish between an interaction between the NND and period and an interaction between year group and school. Also seasonality was not accounted for in the data analysis. Another limitation of the study was that the eating environment when eating packed lunches was not similar to that of the NND meals, thus favouring the NND meals (longer lunch breaks and nicer eating environments when having the NND compared with packed lunches). On the other hand, packed lunches could also have been affected by the parents/children knowing of the measurement being performed. In the present study total food intake (g), edible plate waste (g) and the relative plate waste (compared with the total amount of served food) were analysed whereas the nutrient content of the meals eaten were not analysed. The dietary effects of the NND were evaluated in a separate paper by Andersen *et al.*^(^^30^^)^. The purpose of the OPUS School Meal Study was to test the effect of the NND on multiple outcomes; and the study was designed so the meals were free of charge for the participating children. In a real-life setting a school meal programme will not be free. Another paper will address the cost of the OPUS School Meal Study.

Some strengths of the study are the study design, being a cluster-randomised cross-over design, and the use of advanced statistical analyses taking advantage of the current cluster-randomised cross-over design in a two-step logistic regression model brings convincing results giving the study high power; furthermore, the assessments were taken during a period of 5 d on the same children. Another strength is that the measurements were taken after 3 months of having the NND meals, giving time for the children to get used to the NND meals, which were very different from the usual packed lunch from home. The present study demonstrates convincingly that school children 8–11 years of age are willing to consume more meals that may be unknown to them than described in the literature. We consider that the use of liking measurements of whole meals is novel – and a step forward from the liking measurements of individual foods when taking a public health nutrition perspective and the liking rating is effective as the food intake was 59 % larger when the meals were rated really good compared with when the lunch was rated bad or really bad.

Finally, plate waste studies that weigh foods at the beginning and end of meals are considered to provide detailed, accurate information^(^^15^^)^.

### Conclusion

In conclusion, this cross-over school intervention study showed that the children consumed a significantly higher amount of food at lunch time during the NND period compared with the period on packed lunches from home. The average edible food waste was significantly higher during the NND period compared with the packed lunch period looking at the absolute amounts, whereas the difference in percentage waste was not statistically significant for the NND and packed lunch for meals having plate waste. The study showed that the children's likings of the school meals were inversely associated with the edible plate waste, indicating that likings are essential in attempts to reduce edible plate waste in this age group. Even though some plate waste is inevitable in a school setting, the present study suggests that careful measuring of plate waste together with knowledge of the children's likings can be used to form new strategies to reduce plate waste and getting children to eat nutritious school meals in an economic and environmentally sustainable way.
